# Deciphering the resistance mechanism to *Fusarium* wilt and stem rot of *Passiflora maliformis* var. *pubescens* using histopathology aspects

**DOI:** 10.3389/fpls.2025.1635702

**Published:** 2025-10-07

**Authors:** Daicy Galvis-Tarazona, María Bohórquez-Quintero, Diana Arias-Moreno, Zaida Ojeda-Pérez

**Affiliations:** ^1^ Research Group BIOPLASMA-UPTC, Faculty of Sciences, Universidad Pedagógica y Tecnológica de Colombia, Tunja, Colombia; ^2^ Plant Molecular Biotechnology Laboratory, Division of Molecular Biology, Instituto Potosino de Investigación Científica y Tecnológica AC, San Luis Potosí, Mexico; ^3^ Research Group in Biotechnology and Agricultural Production, Faculty of Agronomy Engineering, Plant Production and Health Department, Universidad del Tolima, Ibagué, Colombia

**Keywords:** Fusarium wilt, stem rot, *Passiflora maliformis*, histopathology, disease resistance, rootstock potential

## Abstract

**Introduction:**

*Passiflora maliformis* is a wild genotype of passionflower with antimicrobial activity and broad phytochemical properties, highlighting its potential as a genetic resource for crop improvement. Given that commercial passionflowers are severely affected by vascular wilt and stem rot caused by *Fusarium oxysporum* and *F. solani*, this study aimed to elucidate the resistance mechanisms of *P. maliformis* var. *pubescens* against these pathogens, with emphasis on histopathological aspects.

**Methods:**

Pathogenicity tests were performed on seedlings germinated *in vitro* and *ex vitro* (SI and SE). Survival, disease incidence, and severity were evaluated in inoculated and non-inoculated plants. In addition, histopathological analyses of roots and stems were performed to characterize structural changes, defense responses, and pathogen colonization.

**Results:**

The genotype exhibited high survival rates and reduced incidence and severity of Fusarium wilt and stem rot, with minimal damage to internal and external tissues. Comparable shoot and root development was observed between inoculated and control plants, indicating the effectiveness of defense mechanisms that maintained physiological performance. Histopathological examination revealed reinforced cell walls, limited pathogen penetration, and restricted vascular colonization.

**Conclusion:**

*P. maliformis* var. *pubescens* demonstrated high responsiveness against *F. oxysporum* and *F. solani*, supporting their role as a potential resistant rootstock. These findings underscore the value of this wild genotype as a strategic genetic resource for breeding programs, integrated disease management, and the sustainable improvement of commercial passionflower crops.

## 1 Introduction

The genus *Passiflora*, comprising more than 520 recognized species, its a taxonomic group of substantial ethnobotanical, horticultural, medicinal, and economic importance (Moraes et al., 2020; Ożarowski and Karpiński, 2021). Its cultivation is predominantly concentrated in tropical and subtropical regions, with Brazil, Colombia, Peru, and Ecuador standing out as the main producers in South America (Fischer et al., 2021). Notably, Colombia is acknowledged as one of the principal centers of genetic diversity, as its heterogeneous agroecological conditions provide optimal environments for the cultivation and diversification of multiple species (Martínez et al., 2020).

In Colombia, the commercial cultivation of passionflowers encompasses taxa of significant agronomic, nutritional, and economic importance, including yellow passion fruit (*Passiflora edulis* f. *flavicarpa* Degener, locally known as maracuyá), purple passion fruit (*P. edulis* f. *edulis* Sims, gulupa), sweet granadilla (*P. ligularis* Juss., granadilla), banana passion fruit (*P. tripartita* var. *mollissima* [Kunth], curuba), and stone granadilla (*P. maliformis* L., cholupa) (Fischer et al., 2021). As of 2024, passion fruit cultivation in Colombia covered approximately 32,430 hectares across 28 departments, yielding an estimated production of 449,775 tons (Ministerio de Agricultura y Desarrollo Rural (MADR), 2025).

*Passiflora maliformis* is also a species of considerable agronomic interest. Based on morphological and genetic analyses, it is classified within the supersection *Stipulata*, section *Granadillastrum*, and series *Tiliifolia* (Yockteng and Nadot, 2004; Ramaiya et al., 2014). Native to the tropical regions of the Americas, this vigorous vine produces large, pendulous hermaphroditic flowers, which display high self-incompatibility (~95%), and fruits that mature within 50–60 days (Molano-Avellaneda et al., 2020). Its phytochemical profile, characterized by a high content of total phenols and strong antioxidant activity, confers extracts with antioxidant, cytotoxic, and antimicrobial properties (Sabogal-Palma et al., 2016).

Another *Passiflora* species of major agronomic importance in Colombia is purple passion fruit (*P. edulis* f. *edulis*), currently ranked as the third most exported fruit. Its profitability and strong demand in European and Canadian markets have driven a steady expansion of its cultivation (López Plazas, 2020). This product is consumed fresh or incorporated into various culinary preparations due to its characteristic aroma and flavor. Its fruits are rich in carbohydrates, essential minerals (Fe, Cu, P, K, Mg), vitamins (riboflavin, niacin, vitamin C), and bioactive compounds with recognized nutraceutical and industrial applications (Fonseca et al., 2022; Pereira et al., 2023). Altogether, these characteristics consolidate purple passion fruit as a strategic crop for Colombia’s agricultural sector and a benchmark for the development of other *Passiflora* species.

Despite their growing socioeconomic relevance, cultivated passionflower species are severely affected by soilborne pathogens, notably *Fusarium oxysporum* and *F. solani*, which cause vascular wilt and stem rot (Fischer and Rezende, 2008; Kiptui et al., 2020; Mukoye et al., 2022; Bernal-Moreno and Rodríguez, 2023; Wang et al., 2023). Under severe infections, these diseases may result in total crop losses (100%) (Torres et al., 1999; Salazar-Gonzalez et al., 2022). Given their persistence in soil and aggressive colonization strategy, these pathogens represent major constraints for purple passion fruit cultivation worldwide, raising significant concerns due to their economic impact (Fischer and Rezende, 2008; Yadeta and Thomma, 2013; Aiello et al., 2021; Kashyap et al., 2021; Gonçalves Barros Silva, 2023).

The infection cycle of *Fusarium* spp. comprises root adhesion, cell wall degradation through enzymatic activity, and xylem colonization by microconidia. These events trigger systemic dissemination that culminates in vascular blockage, physiological water stress, and host death (Ekwomadu and Mwanza, 2023). *Fusarium* wilt and stem rot progress acropetally and manifest through early chlorosis, wilting desiccation of aerial tissues, defoliation, vascular discoloration, and plant death (Ortiz et al., 2014). Stem rot is further characterized by necrosis at the root collar and development of perithecia, although its diagnosis is often complicated by overlapping symptoms with *Fusarium* wilt (Agrios, 2005; Ploetz, 2005).

Managing vascular wilt pathogens is particularly challenging due to their long persistence in soil and their capacity to infect plants at any developmental stage. Conventional control methods, including chemical, physical, and cultural practices, are often ineffective and economically unsustainable (Aiello et al., 2021; Bahadur, 2022; Ekwomadu and Mwanza, 2023). Consequently, increasing emphasis has been placed on genetic strategies such as breeding and grafting with resistant genotypes, which provide sustainable alternatives for the management of *Fusarium* infections (Panth et al., 2020; Carvalho et al., 2021). Grafting, in particular, has been extensively applied in agriculture to enhance crop tolerance to both biotic and abiotic stresses (Hurtado-Salazar et al., 2021; Williams et al., 2021).

While both grafting and breeding provide valuable opportunities for disease management, their application has not been fully extended to all commercial crops, partly due to genetic variability in the response of passion fruit species to pathogens such as *Fusarium* (Ferreira et al., 2023). In this context, studies conducted in producing countries such as Brazil and Colombia, have evaluated the resistance to *F. oxysporum* and *F. solani* of some passion fruit species grown in greenhouses or nurseries (Londoño, 2012; Silva Flores et al., 2012; Ortiz et al., 2014; Forero et al., 2015; Papadaki et al., 2017; Rios Ribeiro et al., 2018; Patiño Pacheco, 2020; Miguel-Wruck et al., 2021).

On the other hand, although *P. maliformis* has been proven to exhibit high compatibility and potential as a rootstock for purple passion fruit (*P. edulis f. edulis*), systematic evaluations of its resistance to *Fusarium* spp., particularly under controlled conditions, remain scarce (Betancourt and Muñóz, 2012; Miguel-Wruck et al., 2021; Roncatto et al., 2021). Therefore, the present study aimed to elucidate the resistance mechanisms of *Passiflora maliformis* var. *pubescens* against *F. oxysporum* (Fusarium wilt) and *F. solani* (stem rot), with emphasis on histopathological traits. The results provide a foundation for enhancing disease resistance and productivity in passionflower crops, supporting the validation of this wild genotype as a resistant rootstock and a valuable candidate for breeding programs.

## 2 Material and methods

This study was carried out at the BIOPLASMA-UPTC Plant Tissue Culture Laboratory in four main phases: (I) *ex vitro* and *in vitro* propagation of *Passiflora maliformis* var. *pubescens* seedlings, (II) obtaining pathogenic isolates of *F. oxysporum* and *F. solani* complexes, (III) inoculation of plant material and response evaluation, and (IV) histological analysis.

### 2.1 Plant and fungal material

Healthy seeds of *Passiflora maliformis* var. *pubescens* from the department of Boyacá were selected as source material. Such seeds were sown under *ex vitro* in peat and maintained under greenhouse conditions (SE) or germinated *in vitro* by zygotic embryo culture (SI) to obtain seedlings. When the SE seedlings were 3–4 cm tall, with development of cotyledonary leaves and a second pair of leaves, they were potted in 0.5 kg bags with a sterile substrate mixture composed of soil:capote:sand in a 3:2:1 ratio.

On the other hand, the SI seedlings were obtained with the methodology reported by Bohórquez et al., (2016). Thus, *in vitro* cultures were established from sexual embryos, to obtain 3–4 cm tall seedlings with developing cotyledonary leaves and a second pair of leaves that were gradually acclimatized in a greenhouse for about 20 days.

Meanwhile, the *Fusarium oxysporum* isolate used in this study was obtained from plants of *Passiflora edulis* Sims f. *edulis* (Gulupa) crops affected by *Fusarium* wilt, established in the municipality of Sutamarchán (Pedregal Bajo; altitude 2.200 m), department of Boyacá, Colombia. Isolated colonies were morphologically characterized from cultures grown on PDA (potato dextrose agar). Proliferated colonies were examined under a stereomicroscope. In addition, microscopic structures were observed on fresh samples stained with lactophenol cotton blue with an optical microscope (Nikon eclipse T2000). Qualitative descriptors, as proposed by Leslie and Summerell (2006) and Sañudo (2012) were used.

Molecular identification was performed by Corpogen Corporation by amplification of the ITS region using the universal primers ITS 4 and 5 (White et al., 1990). The sequence (Accession: PRJNA1216561) was analyzed with the NCBI database using BLAST software. The corresponding fungal isolate was assigned to the species name after comparison with representative sequences available in GenBank and UNITE. Molecular procedures allowed us to obtain fragments of 571 bp as a result of the amplification of PCR products of ITS regions. The taxonomic analysis of this sequence in relation to the NCBI and UNITE nr/nt database indicated a high similarity with *Fusarium oxysporum* (99% identity in 100% of its length).

On the other hand, the isolate of *F. solani* used here (MV184), was previously characterized and identified by Patiño Pacheco (2020) as a causal agent of *Fusarium wilt* in sweet granadilla and also recognized as highly pathogenic for 18 accessions of *Passiflora*.

### 2.2 Pathogenicity test

Two *Fusarium* isolates pathogenic to passion fruits were selected for this study. *In vivo* pathogenicity was tested using two inoculation methods: 1) root immersion and 2) soil infestation with rice grains. The conidial suspension was prepared following the methodology by Namiki et al. (1994) with a concentration of 1x10^6^ con/ml. Seedlings were removed from the substrate, and their roots were washed with sterile distilled water. For inoculation, the root system of uniformly appearing plants was immersed in the conidial suspension for 5 minutes; the solution was agitated before and during the procedure. Then, seedlings were transplanted into a sterile substrate composed of soil, rice husk, and peat in a 3:1:1 ratio.

For soil infestation with the rice grain method, Chaff-Grain medium was prepared following the methodology described by Leslie and Summerell (2006). This suspension was homogenized in 1 kg of substrate as above and the seedlings were sown in the inoculated substrate.

In the pathogenicity assays, several SE and SI seedlings equivalent to those used in each inoculation treatment were included as controls. The roots of these control seedlings were initially immersed in sterile distilled water and then planted in the sterile substrate, following the same procedure described for the inoculated plants.

A completely randomized block design was used with three replications, and an equal number of seedlings was assigned to 10 treatments (**Table 1**). Each experimental unit consisted of six seedlings per replicate. Disease progression was assessed by randomly sampling plants at 3-day intervals, starting from the date of transplanting to pots and continuing for 27 days.

**Table 1:** Inoculation treatments on *Passiflora maliformis* var. *pubescens* germinated *ex vitro* (SE) and *in vitro* (SI), by liquid suspension (L) and infestation on solid substrate (S), with *Fusarium oxysporum* (Fo) and *Fusarium solani* (Fs).

Seedling	Isolate	Inoculation	ID
SE	*Fo*	L	T1-Fo-SE-L
	*Fo*	S	T2-Fo-SE-S
	*Fs*	L	T3-Fs-SE-L
	*Fs*	S	T4-Fs-SE-S
	-	-	T5-C-SE
SI	*Fo*	L	T6-Fo-SI-L
	*Fo*	S	T7-Fo-SI-S
	*Fs*	L	T8-Fs-SI-L
	*Fs*	S	T9-Fs-SI-S
	*-*	-	T10-C-SI

Treatments T5-C-SE and T10-C-SI corresponded to controls; seedling root systems were submerged in sterile distilled water.

### 2.3 Disease assessment.

**Figure 1:**
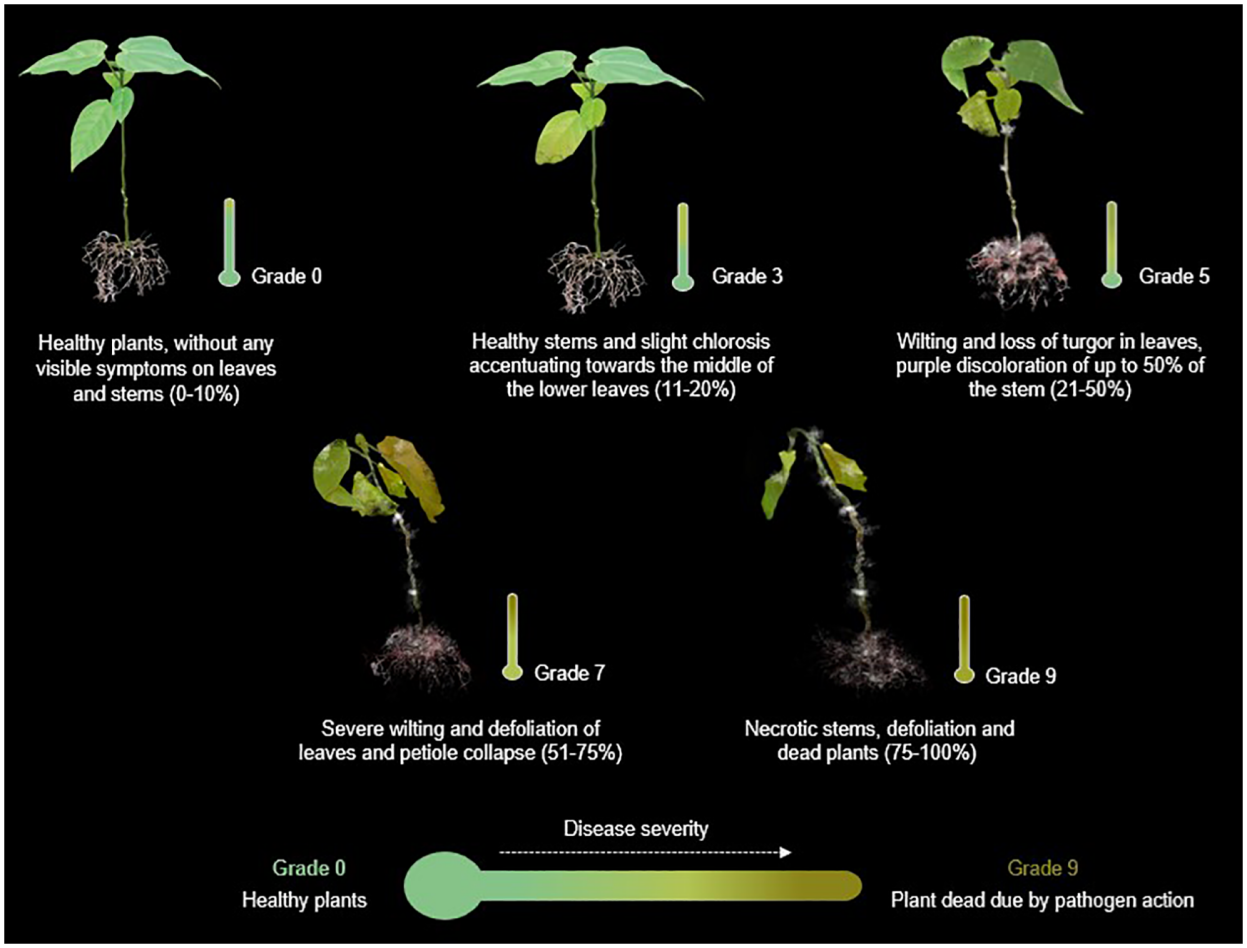
Representative illustration of the progress of *Fusarium wilt* based on symptomatic progression and percentage of the plant affected. Scheme devised and recreated by the authors with images illustrated by Camila Reyes and Angie Carvajal.

The disease progression was assessed through qualitative and quantitative estimators, such as incidence, severity and mortality. *Incubation period* refers to time from inoculation to the expression of disease symptoms in the plants. *Incidence of the disease* was determined by formula: Incidence= (number of diseased individuals/total number of individuals) *100. Regarding *disease severity* was assessed using 0–9 scale based on the presence of discoloration, chlorosis, leaf defoliation, the appearance of the collar and root system, and the percentage of plant area affected (**Figure 1**), as follows: Grade 0 = no infection (0-10%), Grade 3 = 11-20%, Grade 5 = 21-50%, Grade 7 = 51-75%, Grade 9 = 76%- 100%, according to Rodriguez et al. (1999). Seedlings exhibiting severity grades higher than 7 were removed from the pots to further examine the collar and root system.

Moreover, disease intensity was quantified using the severity index, calculated according to the method proposed by Townsend and Heuberger (1943). These values were then used to compute the area under the disease progression curve (AUDPC), which integrates disease development over time for comparison among treatments (López-Santiago et al., 2008). Additionally, *plant mortality* (expressed as a percentage of dead plants), was recorded from each treatment 27 days after inoculation.

### 2.4 Histological analysis

A comparative study of the infection process by both pathogens in the plant tissues was carried out using light microscopy to understand the histological changes/mechanisms associated with the response of *P. maliformis* var. *pubescens* to inoculation with *F. oxysporum* and *F. solani* species complexes. After monitoring, stems and roots were isolated from inoculated plants and SE and SI controls. Tissue samples were sectioned and processed according to the protocol described by Ortiz et al., (2014) with some modifications. The sections were isolated and fixed for 24 h in FAA. Subsequently, these were dehydrated in a series of ethanol isopropanol and acetone. The samples were embedded in paraffin, and histological sections (8-10 µm) were obtained with a rotary microtome. Cross-sections were stained with Safranin-FastGreen and observed under a microscope LEICA DM750 binocular microscope.

### 2.5 Data analysis

Logistic regression analysis was selected as the primary method for data analysis, given the inclusion of multiple independent variables, which justified the use of a multivariate approach. Using a maximum likelihood estimator, the model identified the set of coefficients that maximized the probability of reproducing the observed data. Incidence and severity were coded as binary (“no” = 0, “yes” = 1) and ordinal (scale 0 to 9) variables, respectively, making probabilistic modeling appropriate. A binomial logistic regression model was applied for incidence, while a multinomial logistic regression model was used for severity.

Backward stepwise selection was employed to assess the significance of the factors: day, plant material, fungal species, inoculation technique, and their interactions. Model significance, main effects, and interactions were evaluated using the Chi-square test. Factor levels were compared through odds ratios and predicted probabilities. The AUDPC was analyzed using analysis of variance (ANOVA) to detect significant treatment effects. All statistical analyses were performed using SAS software (SAS Institute Inc, 2019).

## 3 Results

### 3.1 Disease, incidence and severity

**Figure 2:**
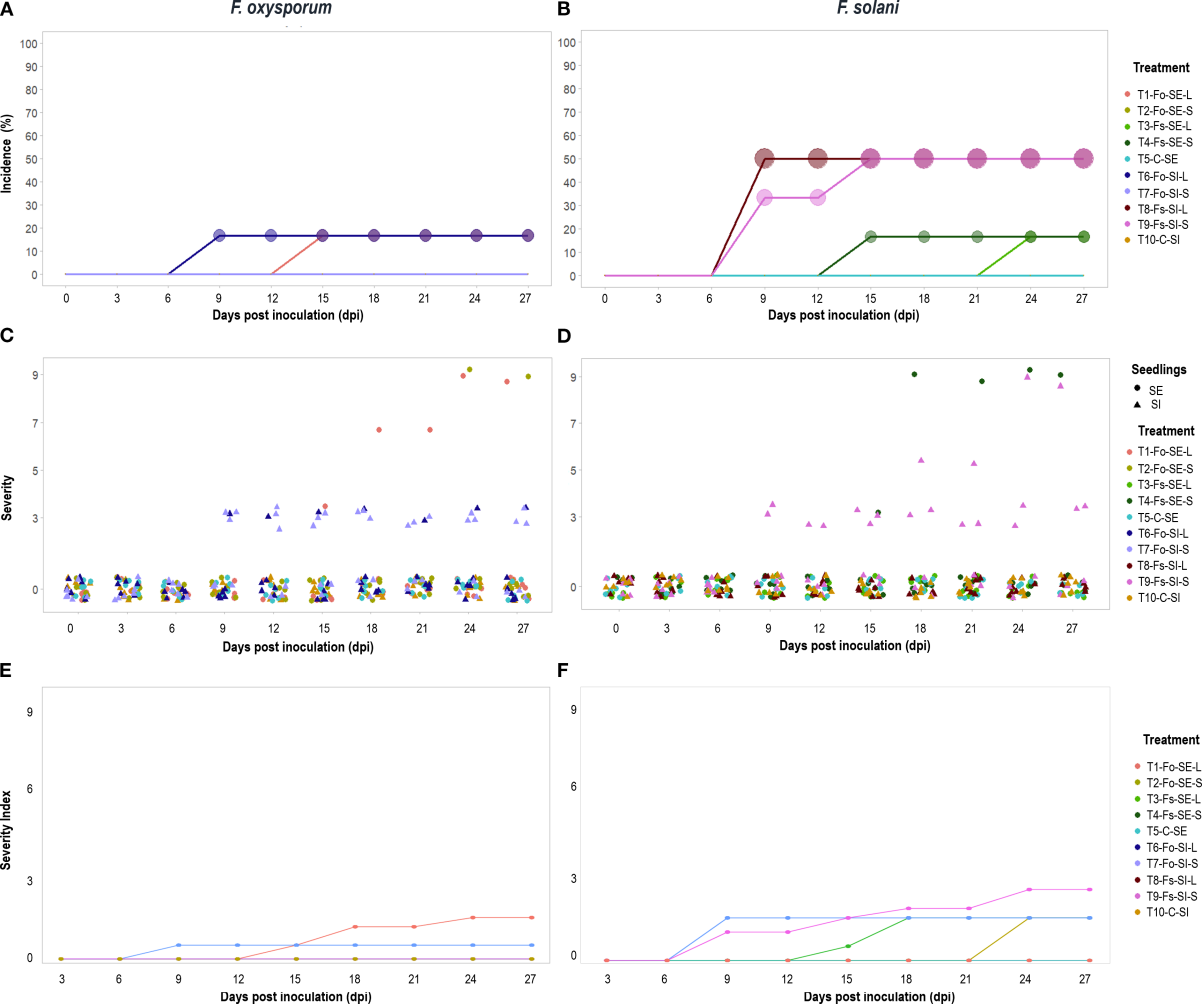
Response of *Passiflora maliformis* var. *pubescens* to *F. oxysporum* (right plots) and *F. solani* (left plots) inoculation during 27 dpi (days post inoculation). In terms of incidence **(A, B)**, the percentage of affected plants coincides with the size of the circles, the higher the value, the larger the size of the figure. Severity **(C, D)**, data set and follow-up to seedlings that integrated the sample and severity index **(E, F)**.

*Symptoms* of *Fusarium* wilt and stem rot after inoculation included yellowing of lower leaves. Then, chlorosis spread through the plant, and the tissues progressively became necrotic and collapsed, ultimately dying. In other cases, symptoms were observable until plants collapsed without prior occurrence of discoloration and disease severity varied among the seedlings evaluated. The incubation period of *F. solani* and *F. oxysporum* was nine days in SI, and 15 days in SE, with both types of inoculation (**Figures 2A, B**).

The *Fusarium* species tested provoked visible symptoms on both SE and SI plants with both inoculation methods. The *incidence* of disease due to *F. oxysporum* after 27 dpi ranged from 0 to 16.67%, and the highest value in both SE and SI plants was recorded with liquid inoculum (**Figure 2A**). With *F. solani*, the percentage rates of infection at 27 dpi ranged between 16.67 and 50%, with the highest data for SI for both types of inoculation (**Figure 2B**).

In terms of *severity*, symptomatic and asymptomatic plants were identified at 27 dpi. Of the diseased plants 60% were slightly affected (grade 3) while the remaining 40% developed drastic symptoms (grade 9) (**Figures 2C, D**), with an F*. solani*: *F. oxysporum* ratio of 5:1 and 3:1 for each grade, respectively. Thus, in SI plants, there was a higher risk of developing the disease and with more severe symptoms under the action of *F. solani* (**Figure 2D**).

The analysis identified statistically significant differences in incidence and severity between fungal species and plant material (SE, SI) (p<0.0001; **Supplementary Tables S1, S2**). In addition, the probability of finding healthy plants of SE and SI was higher than 50%. Likewise, the occurrence of *Fusarium* wilt with medium-high severity (5-9) was less than 10% for both cases (**Supplementary Figure S1**).

The *severity index* obtained was 2.5 on the scale with a maximum value of 9. In plants inoculated with *F. oxysporum* the index was less than or equal to 1.5, while with *F. solani* it was 2.5 (**Figure 2E, F**). In addition, the analysis identified statistically significant differences in severity index between fungal species and plant material (SE, SI) (p<0.001; **Supplementary Tables S3, S4**).

The *AUDPC* obtained ranged from 0 for plants without symptoms to 32.75 for the most affected plants (**Table 2**). The results were not statistically different between treatments (p=0,3263); **Supplementary Table S5**), this can be associated, with low fungal affectation at SE and SI. With respect to *mortality* it was determined that *Fusarium* wilt is not a limiting factor for the viability of *P. maliformis* var. *pubescens.* Only 6.67% of the inoculated plants died by 27 dpi, of which 5.25% and 1.75% corresponded to SE and SI, respectively. In general, the inoculated plants remained viable (93.33%).

**Table 2:** Fusarium wilt AUDPC, 27dpi of Passiflora maliformis var. pubescens with Fusarium oxysporum (Fo) and Fusarium solani (Fs).

Treatment	*F. oxysporum*	*F. solani*
T1-Fo-SE-L	15.25	-
T2-Fo-SE-S	6.75	-
T3-Fs-SE-L	-	0
T4-Fs-SE-S	-	17.25
T5-C-SE	0	0
T6-Fo-SI-L	9.75	-
T7-Fo-SI-S	29.25	-
T8-Fs-SI-L	-	0
T9-Fs-SI-S	-	32.75
T10-C-SI	0	0

### 3.2 Histological analysis of *Fusarium* infection in *P. maliformis* var *pubescens*

**Figure 3:**
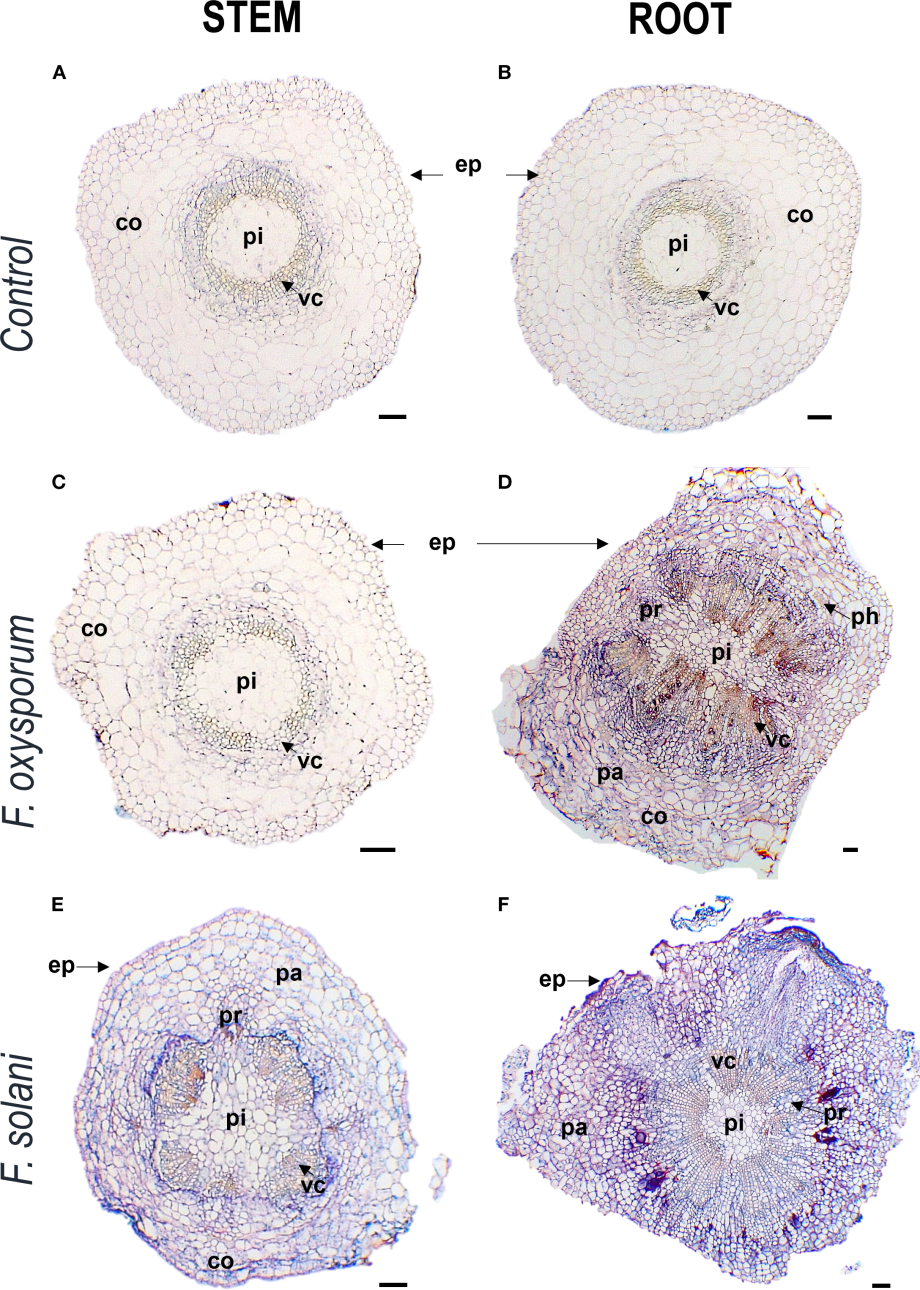
Photomicrographs of cross-cutting sections of stems and roots of *Passiflora maliformis* var. *pubescens* control **(A, B)** and under inoculation with *Fusarium oxysporum* **(C, D)** and *Fusarium solani* **(E, F)**. co, cortex; ep, epidermis; pa, parenchyma; ph, phloem; pi, pith; pr, pith radial and vc, vascular cells. Scale bars, 100 µm. Image created by the authors. The coloration in each image reflects the affinity of the tissues to the Safranin-FastGreen staining method.

In transverse microsections of stem and root of non-inoculated plants, monostratified epidermis, collenchyma, parenchyma, vascular cells, pith and sporadically druses were observed. Vascular cells were arranged in eustella, that is, distributed in a circle and separated by the interphasicular cambium. These tissues and characteristics were also apparent in plants inoculated with *F. oxysporum* and *F. solani*, with some changes (**Figure 3**).

**Figure 4:**
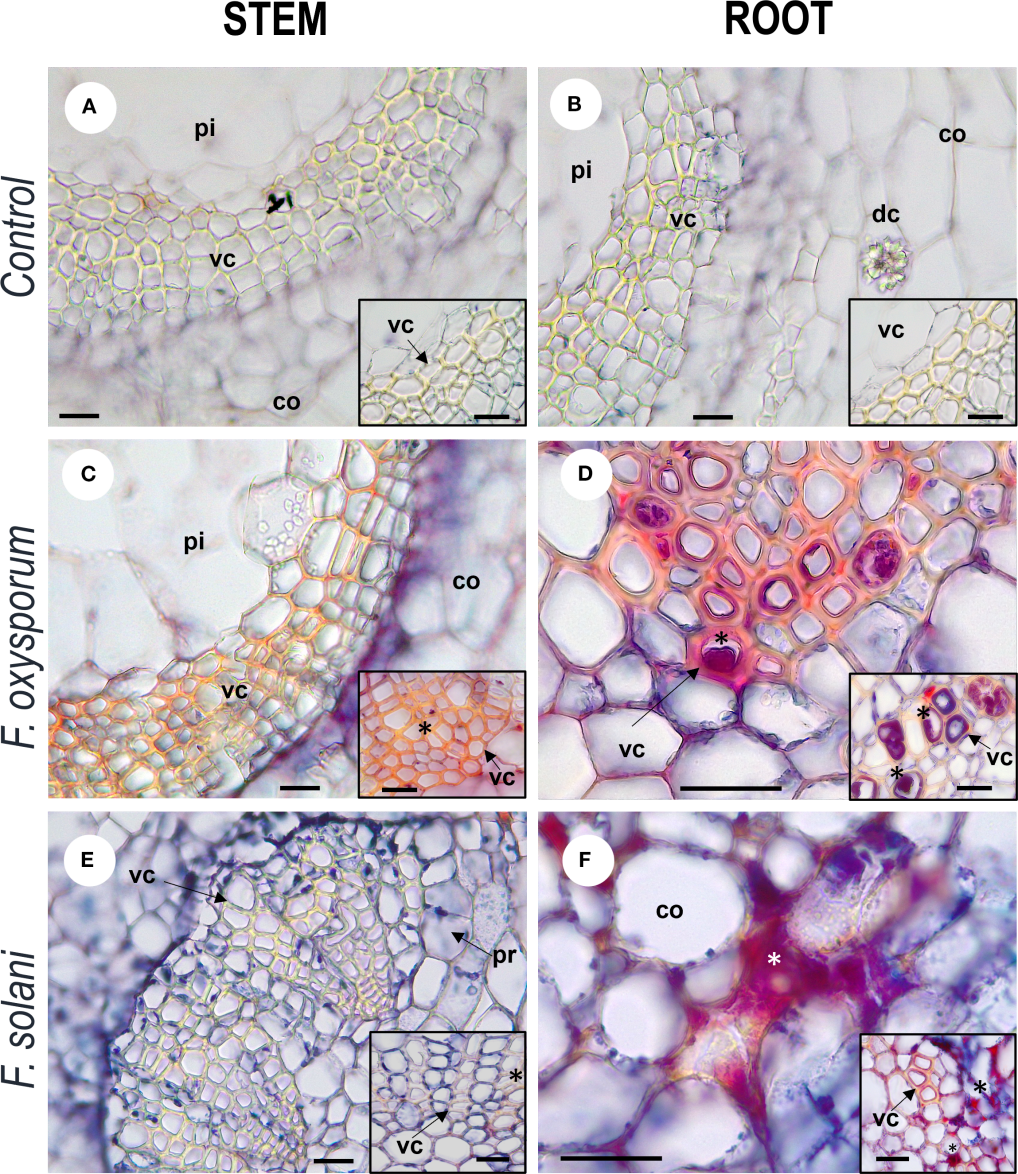
Photomicrographs of cross-cutting sections of stems and roots of *Passiflora maliformis* var. *pubescens* control **(A, B)** and under inoculation with *Fusarium oxysporum* **(C, D)** and *Fusarium solani* **(E, F)**. co, cortex; dc, druse crystal; pi, pith; and vc, vascular cells. Scale bars, 25 µm. Image created by the authors. In inoculated tissues, inside and between the vascular cells there are red-violet dense substances (undetermined), marked with asterisks; absent in the micro-preparations of control plants.

**Figure 5:**
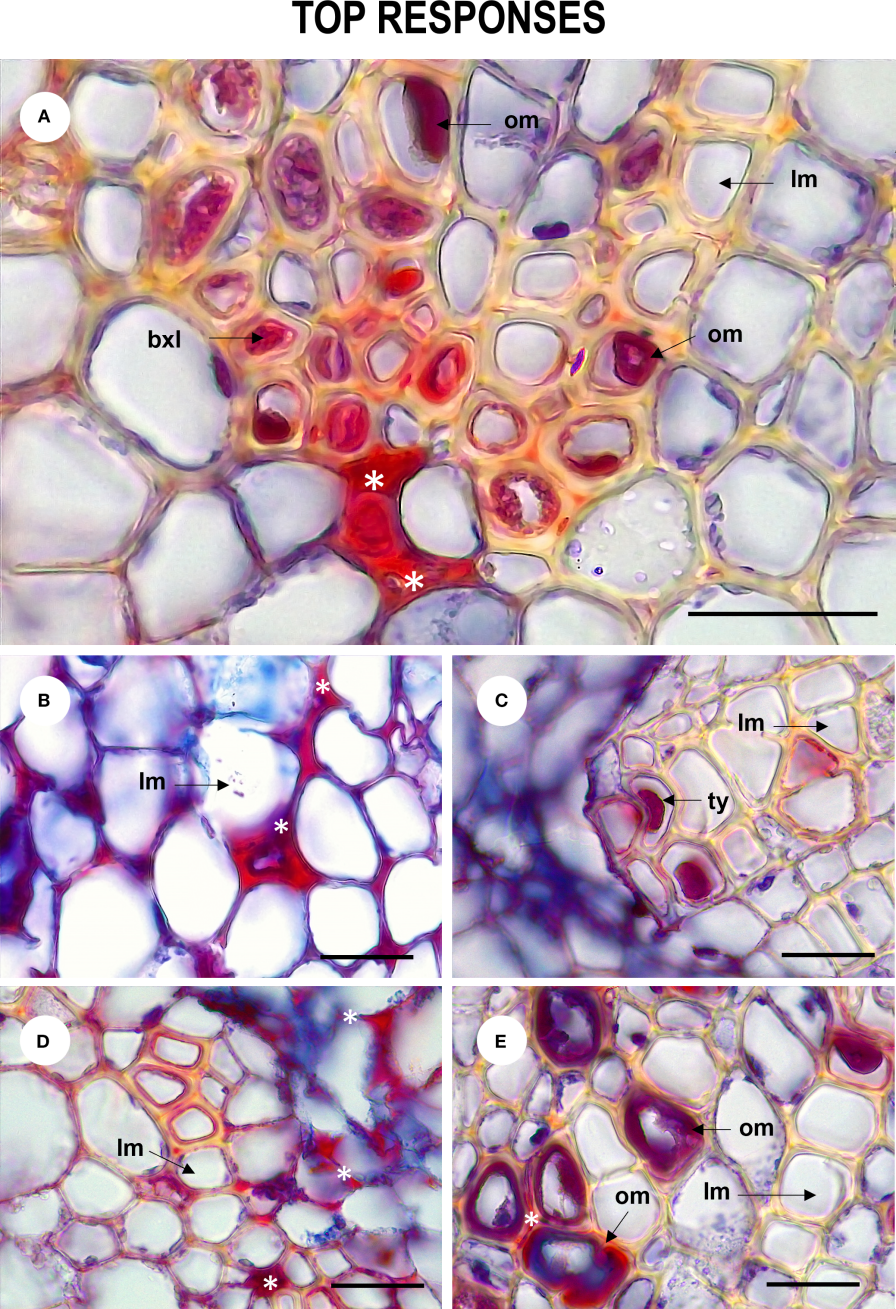
Tissue defense responses of *Passiflora maliformis* var. *pubescens* inoculated with *Fusarium oxysporum* and *Fusarium solani* denoted by the occurrence of dense substances (undetermined) **(A, B, D)**, occluding material **(A, E)** and tyloses **(C)**. om, occluding material; lm, lumen; bxl, blocked xylem lumen and ty, tyloses. Scale bars, 25 µm. Image created by the authors. 27 dpi showing accumulation of substances in the intercellular space (white asterisk).

The anatomical response of the plant to contact with the pathogen was consistent with the degree of severity of the disease. In inoculated plants with mild to medium symptomatology (grades 0 - 5), xylem cells with dense secretions inside and with thickened and lignified cell walls were observed in the stem, showing histological changes compared to control individuals (**Figure 4**). In the root, these mechanisms were more frequent and were complemented by secretions in the intercellular spaces and and what appeared to be tyloses (**Figure 5**). On the other hand, in severely affected plants (grades 7 and 9), tissues gradually collapsed until they suffered complete degradation, while the abundance of fungal spores increased (**Supplementary Figure S2**). These cellular changes coincided with the appearance of noticeable symptoms of infection leading to plant death.

**Figure 6:**
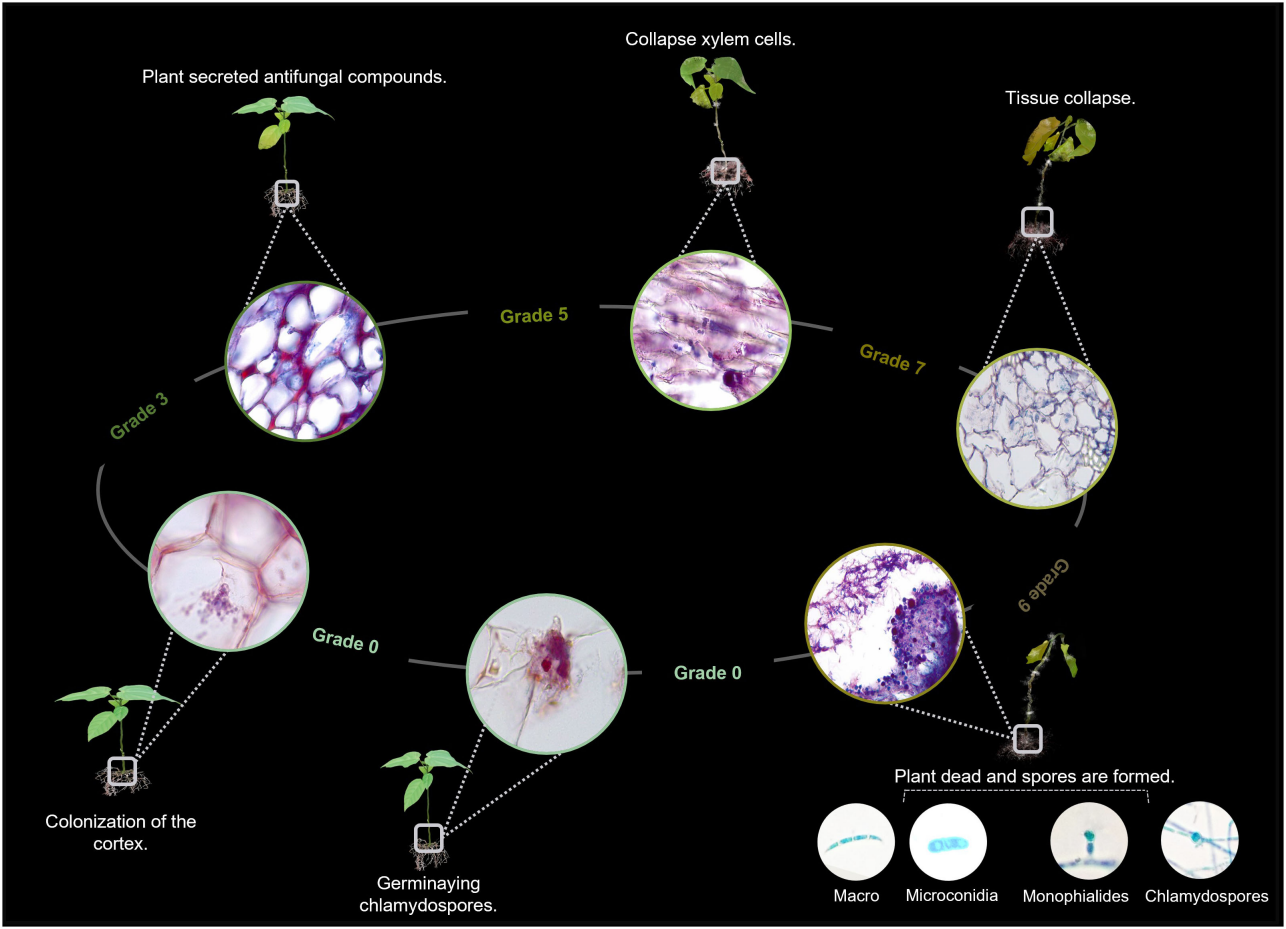
Disease cycle of *Fusarium wilt* in *Passiflora maliformis* var. *pubescens* based on the study conducted; idea adapted from Jangir et al. (2021). The illustration represents the main stages of interaction between the pathogen and the host (plant).

**Figure 7:**
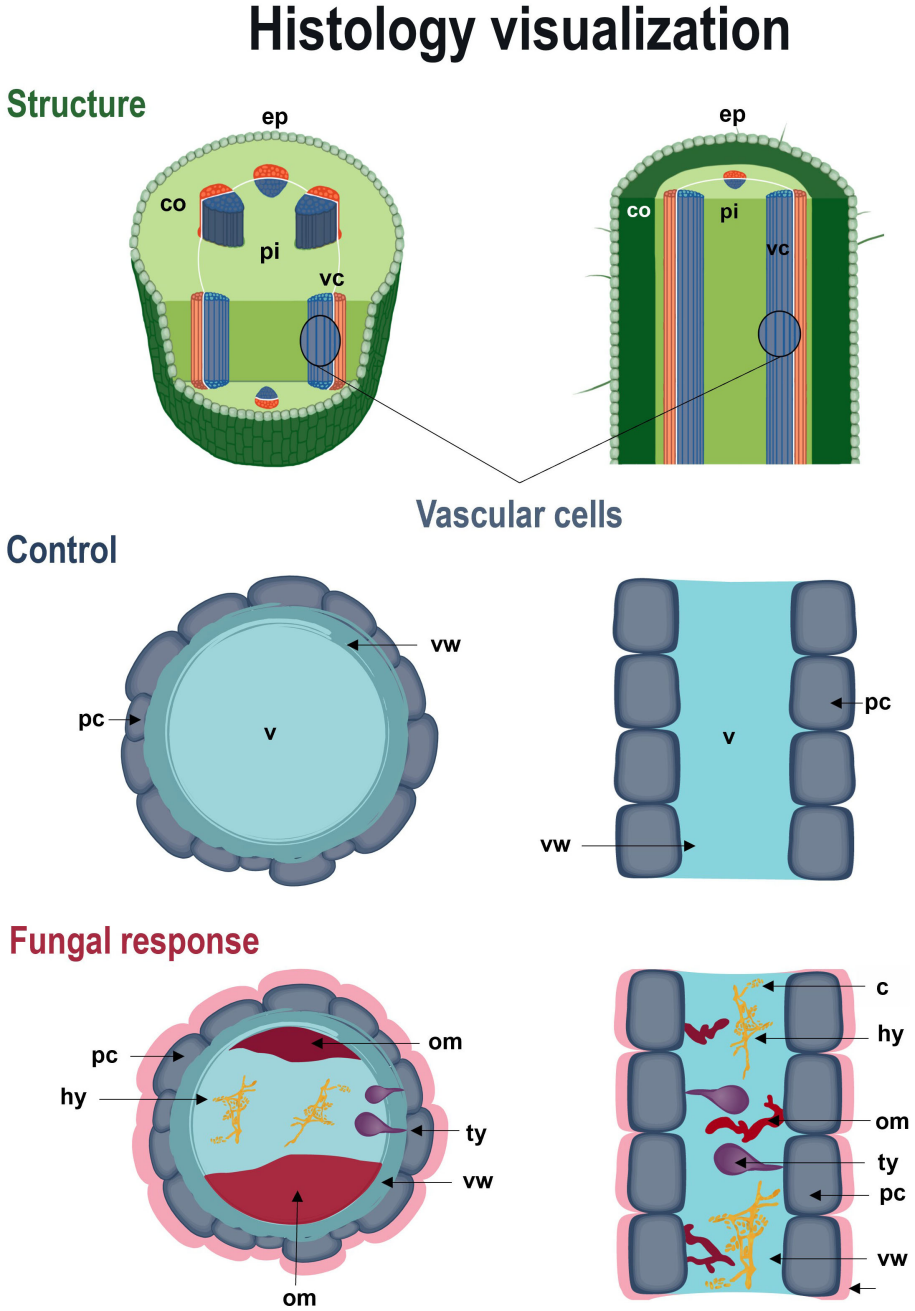
Schematic recreation of defense responses of *Passiflora maliformis* var. *pubescens* to *Fusarium wilt.* Longitudinal and cross-sectional view of the histological response of the host to pathogenic action. co, cortex; ep, epidermis; pi, pith; om, occluding material; sic, substances in the intercellular space; ty, tyloses and vc, vascular cells; v, vessel.

Microscopy allowed to identify and illustrate the main stages of the *Fusarium* wilt disease cycle (**Figure 6**). Thus, it was possible to confirm that the germination of chlamydospores, the development of hyphae that entered the root epidermis and subsequently colonized the cortex constitute the first stages of the disease. Upon colonization, the plant develops response mechanisms to prevent the spread of the fungus in the tissues. However, when fungal infection overcomes these barriers, internal and external tissue collapse occurs in the host, followed by the formation of pathogen reproductive spores (**Figure 7**). Therefore, the results of this study allowed us to relate the activation of defense mechanisms with a lower degree of disease severity.

## 4 Discussion

Research related to diseases affecting crops is essential to guide comprehensive prevention, management and control strategies (Rahut et al., 2022; Zhang et al., 2023). Also, being relevant for the production of safe and healthy food, it can contribute to food security (Li et al., 2023). In the *Passiflora* production sector, it is a priority to have cultivars that are resistant to *Fusarium* wilt and meet nutritional, productive and industrial demands (Amorim-Pereira et al., 2019). However, our current knowledge of the relationship between *F. oxysporum* and *F. solani* and wild genotypes of *Passiflora* is still incomplete. This study contributes to filling that gap, through the evaluation of the response of a wild genotype to the action of *Fusarium* wilt and stem rot causal agents (Mukoye et al., 2022; Nassimbwa et al., 2022). This analysis included aspects frequently reported to determine the response of *Passiflora* species/accessions to the action of *Fusarium* spp., in addition to a histopathological description.

*F. oxysporum* affected less than 17% of the seedlings, with a low symptomatic progression, a severity index of up to 0.5, and an average AUDPC of 6.25. Although inoculation with *F. solani* led to an incidence ≤50%, symptomatology was mild, the severity index was less than 1.75 and disease progress (AUDPC) had a mean value of 21.5. In addition, mortality after 27 dpi in inoculated plants was less than 7%, with similar records for *F. oxysporum* and *F. solani*. These results contrast with those reported for species susceptible to *Fusarium* wilt and stem rot such as *Passiflora edulis* f. *flavicarpa* Degener, *P. edulis* f. *edulis* Sims and *P. ligularis* Juss, where incidence generally ranges between 40-100% (Forero et al., 2015; Melo et al., 2020; Rocha et al., 2021) with medium-high affectation (Ortiz and Hoyos-Carvajal, 2016) and a severity index ranging from 5-7.5 (on a scale of 1-10) (Lima et al., 2019). Likewise, mortality of up to 70% and survival of less than 30% have been reported (Pires et al., 2022).

On the other hand, in the case of resistant accessions, previous studies have reported incidences between 0 and 50% (Londoño, 2012; Patiño-Pacheco and Pérez-Cardona, 2021), mortality rates below 50% (Amorim-Pereira et al., 2019; Melo et al., 2020) survival rates of 67-100% (Carvalho et al., 2021; Oliveira et al., 2022), and progression of severity (AUDPC) close or equal to zero (Pérez and Forbes, 2008); which would indicate a low level of infection in the genotype evaluated in this study.

The search for new sources of resistance against *Fusarium* wilt and stem rot requires efficient detection methods that provide information on physical and chemical response mechanisms (Sampaio et al., 2020). Upon an attack of pathogenic microorganisms plants activate complex immune networks to prevent or minimize colonization of their internal structures and deprivation of nutrients (Margaritopoulou et al., 2020; Duarte-Alvarado et al., 2021). In vascular wilt, the formation of structural barriers in and around the vascular bundles is one of the most important components of defense against the disease according to Kashyap et al. (2021).

Internal anatomical analysis revealed intercellular and intracellular secretion of dense substances, as well as possible lignification of cell walls and tyloses formation, as potential response mechanisms of *P. maliformis* var. *pubescens* to infection by isolates of the *F. oxysporum* and *F. solani* complexes. These reactions play a crucial role in limiting the spread of the pathogen, since physical structures neutralize or reduce further spread and chemicals can lead to the death or inhibition of pathogen growth (De Micco et al., 2016; Chakraborty et al., 2022).

In the initial steps of the response to *Fusarium* wilt, gels are formed within the xylem that are mainly composed of polysaccharides and phenolic compounds exuded from the parenchymal cells adjacent to the infection point. Once these compounds are fused to the cell wall, oxidation and polymerization reactions occur to form a durable barrier at the interface of the infected and healthy tissues (Ardila et al., 2014). Gels and dense substances fix conidia on vascular elements and localize the pathogen when *F. oxysporum* infection occurs (Muche and Yemata, 2022).

Other common barrier mechanisms against the disease are cell wall strengthening due to lignin deposition and the synthesis and accumulation of callose and phenolic compounds in inter- or intracellular spaces that block *Fusarium* progression in the host (Sampaio et al., 2020). Phenols have antifungal capacity by affecting the cell permeability of microorganisms, causing structural and functional changes in enzymes and membrane proteins, leading to alterations in the pH gradient and in the ATP production and conservation system (Carmona et al., 2020).

The results of this study suggested a high response capacity of the genotype since the defense mechanisms limited the pathogenic action of *Fusarium* spp. and low internal and external tissue damage was observed. Overall, both inoculated and non-inoculated SE and SI seedlings exhibited comparable shoot and root growth and development, which may indicate that the defense mechanisms of the genotype were effective in maintaining normal physiological performance. In this sense, the use of *in vitro* culture allowed the rapid and large-scale production of plant material, while providing a platform to evaluate resistance traits under controlled conditions. This dual functionality reinforces its potential application in breeding programs and integrated disease management strategies (Bernal- Moreno et al., 2018; Bernal-Moreno and Rodríguez, 2023). Therefore, these results are especially relevant, as this wild genotype and its genetic background may serve as a strategic resource to address key biotechnological and agronomic challenges currently faced by promising *Passiflora* species.

Given the severe limitations caused by *Fusarium wilt* and stem rot in passion fruits agricultural systems, management of these diseases often relies on synthetic fungicides (Liu et al., 2025). Such as benzimidazoles (e.g., carbendazim), triazoles (e.g., tebuconazole) and strobilurins (e.g., azoxystrobin), which alter fungal membrane integrity or interfere with mitochondrial respiration. However, their continued use has raised increasing concerns about high economic costs, limited systemic mobility, persistence in the environment, risks to human health, and the emergence of resistant pathogen populations (Mikaberidze et al., 2014).

Ecological alternatives for the biorational control of *F. oxysporum* and *F. solani* include fungal and bacterial antagonists such as *Trichoderma harzianum, T. asperellum, T. virens, Bacillus subtilis, B. amyloliquefaciens, Pseudomonas fluorescens, P. putida, and P. protegens.* These microorganisms have demonstrated inhibition rates of up to 94% *in vitro* and, in some cases, in the field, through mechanisms that include mycoparasitism, the production of hydrolytic enzymes (e.g., chitinase and β-1,3-glucanase), the emission of volatile organic compounds, and the induction of systemic resistance in host plants (Verma et al., 2007; Ongena and Jacques, 2008; Ben Khedher et al., 2015; Mehmood et al., 2023; Yao et al., 2023). However, the effectiveness of these strategies in the field remains variable, largely influenced by factors such as interactions with native soil microbiota, climate variability, and the limited persistence of inoculants in the rhizosphere (Awu et al., 2023; Wang et al., 2024).

In this context, the use of resistant rootstocks emerges as a complementary and sustainable solution. Resistant genotypes do not require repeated applications, do not generate toxic residues, and are less susceptible to environmental fluctuations (Ayala-Doñas et al., 2020; Suansia and Chandra Samal, 2021). Therefore, the identification of *Passiflora* rootstocks resistant to *Fusarium* wilt and stem rot represents a significant contribution to Colombia, one of the world’s leading producers and exporters. This strategy offers a low environmental impact alternative that can improve the productivity of commercial species, reduce the incidence and severity of *Fusarium*-related diseases, and promote adaptability in various growing conditions.

## Data Availability

The datasets presented in this study can be found in online repositories. The names of the repository/repositories and accession number(s) can be found in the article/**Supplementary Material**.
