# Supplementary material for: Deciphering the resistance mechanism to Fusarium wilt and stem rot of Passiflora maliformis var. pubescens using histopathology aspects
Source: Front Plant Sci. 2025 Oct 7;16:1635702. doi: 10.3389/fpls.2025.1635702 (PMC12538707; doi:10.3389/fpls.2025.1635702)
Supplement: Supplementary file 1 [file DataSheet1.docx]

Supplementary Material

# Supplementary Figures and Tables

## Supplementary Figures

**Figure S1.** Probability of occurrence of *Fusarium wilt*, according to the severity scale (0-9), in *Passiflora maliformis* var. *pubescens* germinated *ex vitro* (SE) and *in vitro* (SI) non-inoculated (control) and inoculated with *Fusarium oxysporum* and *Fusarium solani*.


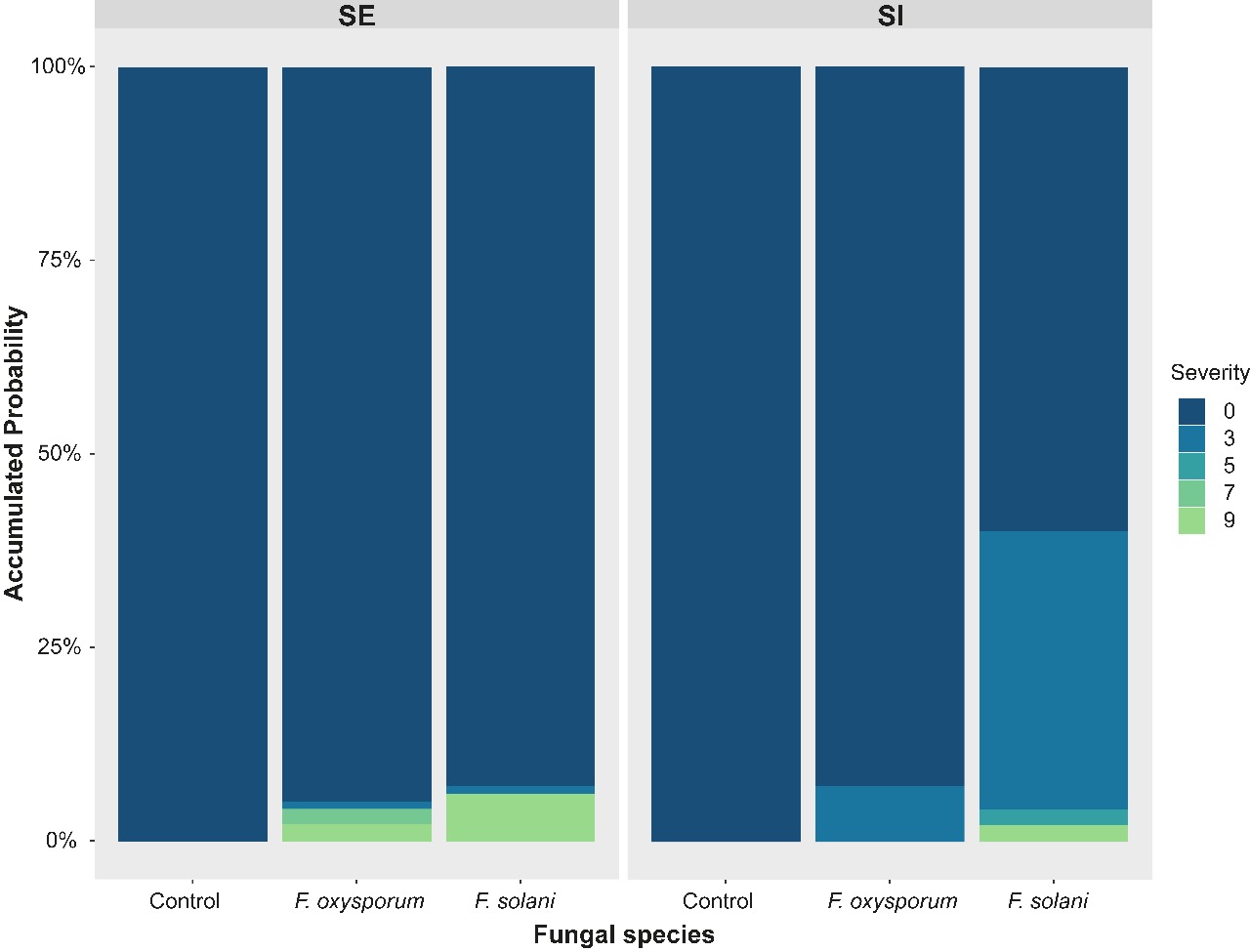


**Figure S2.** Photomicrographs of cross-cutting sections of stems and roots of *Passiflora maliformis* var. *pubescens* severely affected by *Fusarium oxysporum* and *Fusarium solani* and formation of fungal structures of these species. co: cortex, ep: epidermis, c: chlamydospores, cc: collapse cells, hy: hyphae, ph: phloem and vc: vascular cells. Scale bars, 100 µm. Image created by the authors.

**
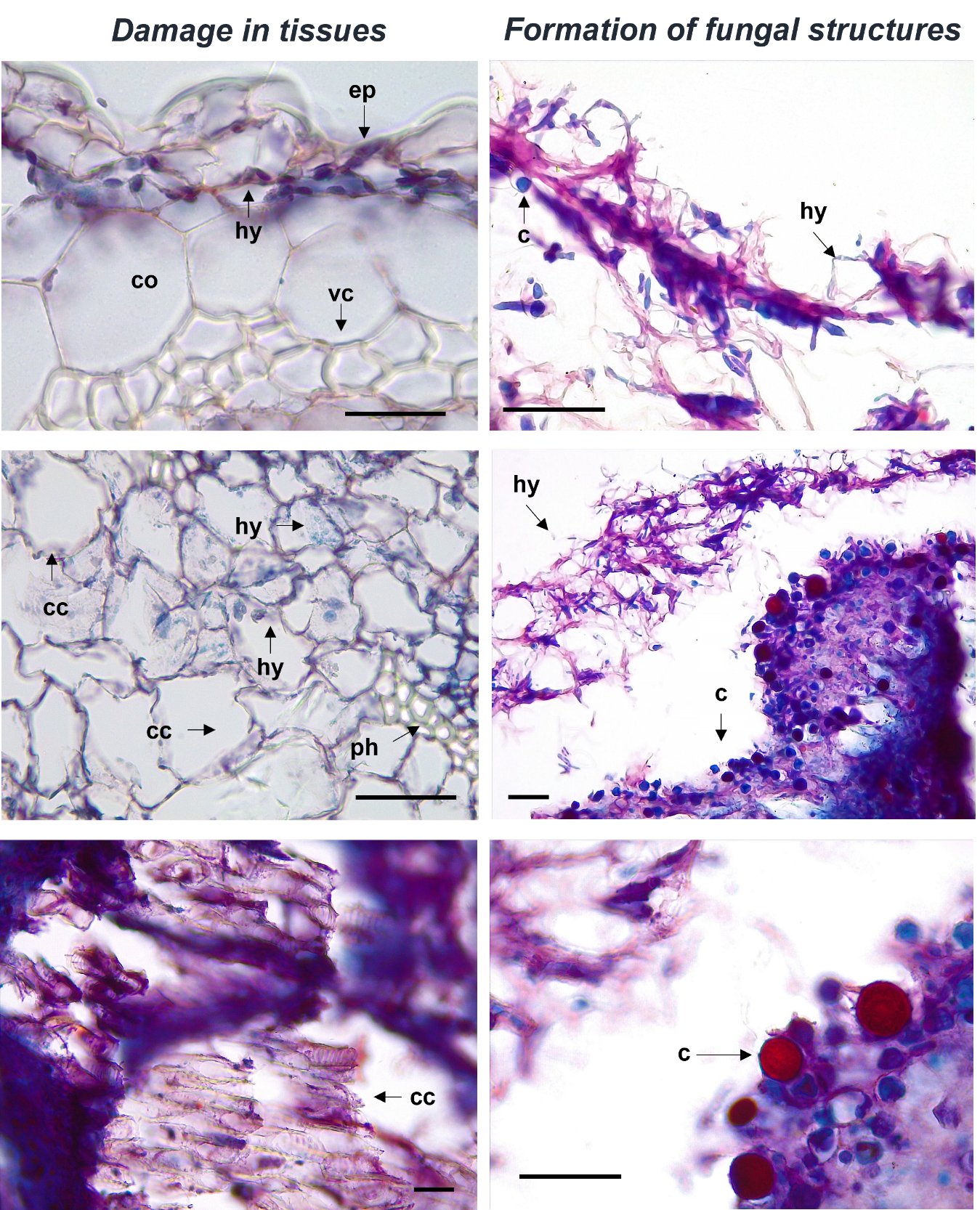
**

## 1.2 Supplementary Tables

**Table S1** Results of logistic regression and backward selection analyses of effect of day, plant material, fungal specie and inoculation technique on *Fusarium wilt* **incidence** in *Passiflora maliformis* var. *pubescens* inoculated with *Fusarium oxysporum* and *Fusarium solani*.

| Step | Effect eliminated | DF | Number in | Chi-cuadrado de Wald | Pr>ChiSq |
| --- | --- | --- | --- | --- | --- |
| 1 | Day *Plant material *Fungal specie *Inoculation technique | 8 | 14 | 0.0038 | 1.0000 |
| 2 | Day *Plant material *Fungal specie | 16 | 13 | 0.0472 | 1.0000 |
| 3 | Day *Fungal specie *Inoculation technique | 8 | 12 | 0.0030 | 1.0000 |
| 4 | Day *Plant material *Inoculation technique | 16 | 11 | 0.4628 | 1.0000 |
| 5 | Day *Fungal specie | 16 | 10 | 0.5186 | 1.0000 |
| 6 | Day *Inoculation technique | 16 | 9 | 0.7378 | 1.0000 |
| 7 | Day *Plant material | 8 | 8 | 0.3901 | 0.9999 |
| 8 | *Plant material *Fungal specie *Inoculation technique | 1 | 7 | 0.0000 | 0.9953 |
| 9 | Day | 8 | 6 | 3.3001 | 0.9141 |
| 10 | Fungal specie *Inoculation technique | 1 | 5 | 0.0254 | 0.8733 |
| 11 | Plant material *Inoculation technique | 1 | 4 | 0.0954 | 0.7575 |
| 12 | Inoculation technique | 1 | 3 | 2.7787 | 0.0955 |
| 13 | Plant material *Fungal specie | 2 | 2 | 5.7256 | 0.0571 |
| 14 | Plant material | 1 | - | 22.2120 | <.0001 |
| 15 | Fungal specie | 2 | - | 22.2174 | <.0001 |

DF: Degrees of freedom

Pr: Probability

**Note**: No (additional) effects met the 0.05 significance level for removal from the model.

**Table S2** Results of logistic regression and backward selection analyses of effect of day, plant material, fungal specie and inoculation technique on *Fusarium wilt* **severity** in *Passiflora maliformis* var. *pubescens* inoculated with *Fusarium oxysporum* and *Fusarium solani*.

| Step | Effect eliminated | DF | Number in | Chi-cuadrado de Wald | Pr>ChiSq |
| --- | --- | --- | --- | --- | --- |
| 1 | Day *Plant material *Fungal specie *Inoculation technique | 8 | 14 | 0.0039 | 1.0000 |
| 2 | Day *Plant material *Fungal specie | 16 | 13 | 0.0472 | 1.0000 |
| 3 | Day *Fungal specie *Inoculation technique | 8 | 12 | 0.0031 | 1.0000 |
| 4 | Day *Plant material *Inoculation technique | 16 | 11 | 0.7039 | 1.0000 |
| 5 | Day *Fungal specie | 16 | 10 | 0.4545 | 1.0000 |
| 6 | Day *Inoculation technique | 16 | 9 | 0.8343 | 1.0000 |
| 7 | Day *Plant material | 8 | 8 | 0.5234 | 0.9998 |
| 8 | *Plant material *Fungal specie *Inoculation technique | 1 | 7 | 0.0000 | 0.9953 |
| 9 | Day | 8 | 6 | 3.8067 | 0.8741 |
| 10 | Fungal specie *Inoculation technique | 1 | 5 | 0.0259 | 0.8722 |
| 11 | Plant material *Inoculation technique | 1 | 4 | 0.0151 | 0.9023 |
| 12 | Inoculation technique | 1 | 3 | 1.9415 | 0.1635 |
| 13 | Plant material *Fungal specie | 2 | 2 | 5.1938 | 0.0745 |
| 14 | Plant material | 1 | - | 19.670 | <.0001 |
| 15 | Fungal specie | 2 | - | 20.8132 | <.0001 |

DF: Degrees of freedom

Pr: Probability

**Note**: No (additional) effects met the 0.05 significance level for removal from the model.

**Table S3** Kruskal-Wallis One-Way table showing the effect of the treatment on *Fusarium wilt* **severity index** in *Passiflora maliformis* var. *pubescens* inoculated with *Fusarium oxysporum* and *Fusarium solani*.

| Factor | Sum square | GL | p |
| --- | --- | --- | --- |
| Average Severity | 259 | 9 | <.001 |

GL: Degrees of freedom

p: Probability

**Table S4** Dwass-Steel-Critchlow-Fligner two-to-one comparisons table showing the effect of the fungal specie and plant material on *Fusarium wilt* **severity** in *Passiflora maliformis* var. *pubescens* germinated ex vitro (SE) and in vitro (SI) inoculated with *Fusarium oxysporum* and *Fusarium solani*.

| Group 1 | Group 2 | W | p |
| --- | --- | --- | --- |
| *F. oxysporum* | *F. solani* | 9.75 | < .001 |
| SE | SI | 6.52 | < .001 |

**Table S5** ANOVA table showing the effect of the treatment on *Fusarium wilt* **progression** in *Passiflora maliformis* var. *pubescens* inoculated with *Fusarium oxysporum* and *Fusarium solani*.

| Factor | DF | Type III SS | Mean Sq |  | F-Value | Pr > F |
| --- | --- | --- | --- | --- | --- | --- |
| Treatment | 9 | 4100.325000 | 455.591667 |  | 1.24 | 0.3263 |

DF: Degrees of freedom

Pr: Probability

**
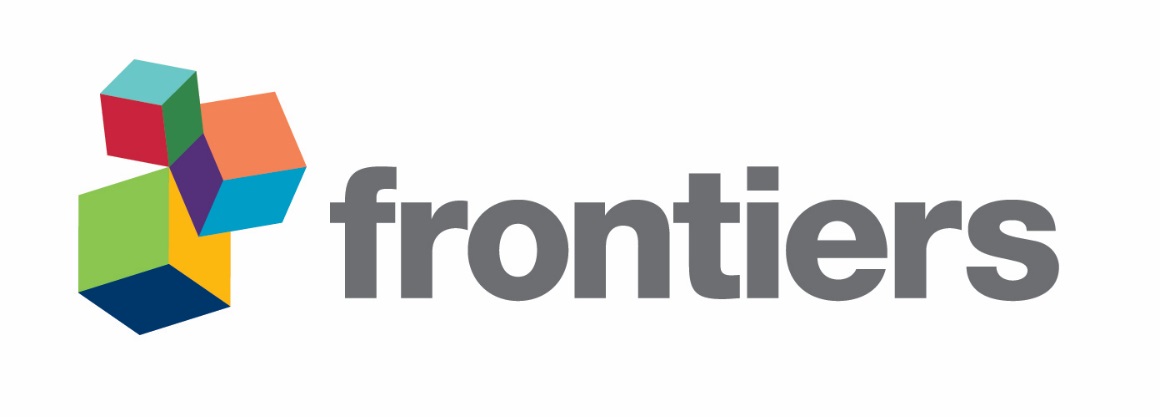
**
